# Decomposing Income Inequality: The Role of the Happiness Gap

**DOI:** 10.3390/healthcare13121401

**Published:** 2025-06-12

**Authors:** Jinxian Wang, Yuzhou Wang, Jianfeng Yan

**Affiliations:** 1 Business School, Central South University, Changsha 410083, China; 2 School of Public Administration and Policy, Shanghai University of Finance and Economics, Shanghai 200433, China; wangyuzhou@163.sufe.edu.cn (Y.W.); yanjianfeng@mail.sufe.edu.cn (J.Y.)

**Keywords:** happiness gap, income inequality, decomposition, CFPS

## Abstract

**Background/Objective:** While positive emotions enhance productivity, little is known about whether income inequality will decrease if low-income individuals become happier. **Methods**: Relying on data from China Family Panel Studies (CFPS) from 2010 to 2018 and a regression-based decomposition method, this study investigates the contribution of happiness gap to income inequality. **Results**: The results show that a 1% increase in happiness leads to a 0.064–0.124% increase in income. This study employs average daily sunshine hours as an instrumental variable for happiness. The results from the two-stage least squares estimation also support the conclusion that an increase in happiness can lead to higher income. The decomposition results show that the happiness gap increases income inequality, although its contribution has decreased between 2010 and 2018. This positive effect is attributed to gaps in physical health and spare time devoted to learning. More precisely, happiness improves physical health among the upper-middle-income group and promotes spare time devoted to learning in the high-income group. Conversely, happiness narrows income inequality by reducing psychological stress in the low-income group. **Conclusions**: The results suggest that the enhancement of residents’ sense of acquisition, satisfaction and happiness, especially among the low-income group, thereby reducing income inequality.

## 1. Introduction

The importance of reducing income inequality has been best featured by the Millennium Development Goals (MDGs), as well as the post-2015 development agenda of the Sustainable Development Goals (SDGs). However, income inequality has been increasing all over the world in the past decades. From the global perspective, in 2021, the global top 10% owns 52% of total household income, while the bottom 50% captures only 8% of the total income, according to the World Inequality Report 2022. Regarding China, it has experienced a remarkable increase in income inequality since its economic reform and opening-up, although there was a slight decrease in the Gini coefficient after 2008. This high and rising inequality has brought several adverse effects on China [1]. So far, many studies have been exploring the forces behind income inequality, among them government policies, labor market changes, and individual characteristics are widely referenced [2,3,4,5,6].

A few scholars have figured out that happiness accounts for changing income and income inequality [7,8,9]. In China, the happiness level of residents has generally increased since 2010 [10]. Based on data from the China Family Panel Studies (CFPS), the proportion of residents who are “very satisfied” with their lives increased from 16.1% in 2010 to 33.6% in 2018, and the proportion of residents who are “dissatisfied” decreased from 10.6% in 2010 to 3% in 2018. The variance of residents’ happiness also decreased from 1.04 in 2010 to 0.96 in 2018. At the same time, the Gini coefficient of income during the same period decreased from 0.485 in 2010 to 0.460 in 2018. Hence, the question arises as whether the narrowing of the happiness gap contributed to the decline in income inequality? If so, what are the transmission mechanisms?

This study adds to the existing literature by answering these questions, relying on data from CFPS for five waves of 2010, 2012, 2014, 2016, and 2018. We first follow Card et al. [11] to examine the causal relationship between happiness and income, using unexpected sunshine hours as the instrument variable. Based on these results, we further use a decomposition method to calculate the contribution of happiness gap to income inequality. After that, we check the influencing mechanisms in different income groups, so as to analyze the effect of happiness on individual income via perspectives of physical health, mental health, and spare time devoted to learning.

The results show that happiness is positively and significantly associated with income. This study also employs average daily sunshine hours as an instrumental variable for happiness. Applying this instrumental variable is because: Firstly, existing literature indicates that climate data, such as daylight exposure, can influence people’s happiness [12,13]; secondly, average daily sunshine hours do not directly affect residents’ income. The results from the two-stage least squares estimation also show that an increase in happiness can raise residents’ income, consistent with the baseline regression results.

Further, it shows that the gap in happiness among different groups of people has contributed to income inequality. Interestingly, this contribution has declined over time. The mechanism analysis suggests that physical and mental health as well as spare time devoted to learning are the pathways. Specifically, happiness increases individual income by improving physical health among the upper-middle-income group and increasing spare time devoted to learning in the high-income group. Conversely, happiness reduces psychological stress in the low-income group, leading to a lower income inequality, which explains the diminishing contribution of the happiness gap to income inequality over time.

Our study sheds light on the existing studies in the following three aspects. First, to the best of our knowledge, this is the first study to calculate the specific contribution of the happiness gap to income inequality. Our decomposition method is based on the Shapley decomposition, referring to Chen et al. [14]. Second, this study adds to our understanding of happiness. The existing literature rarely focuses on the effect of the happiness gap on income inequality or only provides indirect evidence by examining the heterogeneity effect of happiness on income among different income groups [15]. The results of this study show that the happiness gap is in fact an important contributor to income inequality. Third, we study the mechanisms of happiness gap on inequality from the perspectives of physical and mental health as well as spare time devoted to learning [16,17], therefore complementing the literature regarding how the happiness gap may affect income inequality.

## 2. Literature Review

Happiness is a kind of positive emotion, showing that individuals are in a state of full attention, pleasure, and devotion [18,19]. It affects the physical and mental health of individuals, their work life and even the development and stability of society as a whole. Research on happiness and the standard of living of the population can be traced back to Fisher and Hanna (1931) [20]. They argued that workers’ emotional instability leads to maladjustment in their occupations and that poor moods impede success. Later, Diener explored the concept and measurement of subjective well-being in his seminal 1984 article “Subjective Well-being”, positing that happiness is primarily a subjective emotion. Drawing on the perspective of Benjamin et al. (2014) [21], this study utilizes respondents’ subjective ratings of their own happiness as an indicator to measure happiness (in accordance with Benjamin et al. (2014) [21], this paper employs subjective well-being as a proxy for individual happiness; therefore, the two terms are used interchangeably in the subsequent text).

So far, many scholars have conducted research on the impact of happiness or life satisfaction on the income of individuals, organizations, and nations, and most of these studies have shown that happiness promotes income growth [7,8]. Other scholars have found that happiness can improve physical and mental health [22,23], labor productivity [24], social skills [25], and residents’ sense of responsibility [26], which therefore increase the income levels of individuals, organizations, and society as a whole.

### 2.1. The Impact of Emotions on Income Disparities

We searched for published studies and working papers analyzing the effect of happiness on income inequality. We employed search engines including Google Scholar, Research Gate, ISI Web of Science, and Econlit and entered keywords such as “happiness”, “subject well-being” on the one hand, “inequality”, “unequal development” and “income distribution” on the other hand. So far, there is no study directly examining the relationship between happiness and income inequality, while there are only three studies investigating the relationship between emotions (including mental health) on the one hand, economic outcomes on the other hand. Lund et al. (2020) [15], through an experiment of 39 mental health interventions in 36 low- and middle-income countries, find that improvements in the mental health of the population contribute to the improvement of economic level of the country. This effect was stronger in countries with higher income, which was nearly twice as high in the 12 highest-income countries than in the 12 lowest-income countries. Li and Yu (2020) [9] study the impact of happiness on residents’ consumption using CFPS data, noting that the happier the head of the household, the higher willingness to consume. Their mechanistic studies have found that happiness can contribute to an increase in residents’ income by encouraging individuals or households to enrich their social networks, with higher happiness groups experiencing greater income growth. In contrast, negative emotions, such as stress, may worsen income distribution. Camacho (2008) [27], based on data of Colombian newborns from 1998 to 2003, finds that violence-induced psychological stress in pregnant women leads to lower newborn weights. In turn, babies born with lower birth weights have worse disease conditions in adulthood, which, as the author points out, has a negative impact on intergenerational human capital accumulation, thus, exacerbating income inequality.

### 2.2. The Mechanisms Through Which Emotions Affect Residents’ Income

Furthermore, we have reviewed the relevant mechanisms, through which emotions may affect income and income inequality, including the impact of emotions on health, job performance, and cognitive abilities (these mechanisms will be tested in Section 4.4, Mechanism Analysis). Some literature points out that happiness is positively correlated with an individual’s physical and mental health. Cohen et al. (2003) and Appleton et al. (2011) [22,23] conduct studies on 18–55-year-olds, respectively, and find that positive emotions strengthen an individual’s resistance to a number of illnesses. Blanchflower et al. (2013) [16], through a survey study in the UK, find that good mood promotes the development of good living habits, thus, improving physical and mental health. Through a survey of over a hundred workers, Yuliana et al. (2015) [28] argue that increased work stress exacerbates burnout in the workplace, which negatively affects work efficiency. Tenney et al. (2016) [17] also point out that employees with higher happiness not only have better health but also have a better ability to regulate themselves under work stress. Andersson and Harnois (2020) [29], using data from the U.S. General Social Survey (GSS), find that women with low well-being have difficulty overcoming stress from job pressure and gender discrimination. Barker et al. (2021) [25], through an experiment in poor rural households in Ghana, demonstrate that well-being can increase household income levels by reducing depression and enhancing cognitive and social skills. In addition, subjective emotions can also affect motivation to learn. As indicated by Delaney et al. (2014) [30], through a laboratory experiment, residents over the age of 50 would significantly reduce their effort to learn about financial decisions when they are under increased stress.

Some scholars have also found that happiness can influence income levels by affecting personality traits such as patience and conscientiousness. Fry’s (1975) [31] results from an experiment with children show that people who have positive emotions during childhood tend to have better patience. Specht et al. (2013) [26], using data from a German micro-survey, argue that individuals with higher life satisfaction have better emotional stability and dutifulness, and show greater performance when facing with social role transitions (e.g., marriage, childbearing, employment).

Overall, the fact that the effect of happiness on income varies among residents engaged in different industries, or with different cognitive abilities, suggests that there is an effect of the happiness gap on income inequality, which inspires us to explore the mechanisms by which the happiness gap affects income inequality. As such, this study aims to examine the contribution of the happiness gap to income inequality and its influencing mechanisms, which can be estimated through a regression-based decomposition method according to the Shapley theory.

## 3. Model and Data

### 3.1. Model

The aim of this study is to estimate the contribution of the happiness gap to income inequality. This will be estimated in two steps. The first step is to analyze the impact of happiness on individual income by constructing the income determination equation. The second step is to decompose the specific contribution of the happiness gap (or the distribution of happiness) based on the first step [32]. Below, we introduce the two steps in detail.

#### 3.1.1. Income Determination Equation

In the first step, we refer to Card et al. (2013) [11] to specify the income determination equation as follows:

(1) $$\ln\;y_{i}=c+\beta^{\prime }{happiness}_{i}+\gamma_{i}^{\prime }X_{i}+{province}_{i}+\varepsilon_{i}$$

$\ln\;y_{i}$ is the logarithm of income for individual i. ${happiness}_{i}$ is the individual $i^{\prime }s$ happiness. ${province}_{i}$ represents provincial fixed effect. $\varepsilon_{i}$ is the residual term. $X_{i}$ includes a set of control variables. We consider both environmental and effort factors in $X_{i}$ that affect individual income, including gender, age, education, work industry, cognitive ability, household registration type (that is, the hukou system in China), migration, and parents’ education level. $\beta^{\prime }$ and $\gamma_{i}^{\prime }$ are the parameters to be estimated. Since this paper will estimate the contribution of happiness to income inequality in each year under study, the corresponding income determination equation does not include the year fixed effect.

#### 3.1.2. Decomposition of Income Inequality

In the second step, we decompose the contribution of the happiness gap as well as other control variables to income inequality based on the income determination equation above. Especially, the semi-log specification in Equation (1) implies a nonlinear income determination function in terms of the original income variable. We obtain the individual income $y_{i}$ by taking the natural logarithm on both sides of Equation (1) when decomposing the income determination equation:

(2) $$y_{i}=exp\;(\hat{c}+{province}_{i})\times exp\;({\hat{\beta }}^{\prime }{happiness}_{i}+{\hat{\gamma }}_{i}^{\prime }X_{i})\times exp\;({\hat{\varepsilon }}_{i})$$

To conduct a regression-based decomposition, the Shapley value framework is adopted [14,33]. We first calculate the Gini coefficient of income $y_{i}$, $Gini(y_{i})$. Then, all variables of the income generation equation are decomposed separately. For example, in the first-round decomposition, we replace $x_{1}$ by its sample mean ${\bar{x}}_{1}$ to eliminate any differences in $x_{1}$ among individuals. Next, we re-compute income ${\hat{y}}_{1i}$ and the Gini coefficient $Gini({\hat{y}}_{1i}$) using ${\bar{x}}_{1}$ and real values of other variables. By doing so, we can obtain the contribution of variable $x_{1}$’s distribution (or inequality) to income inequality: $Gini\left(x_{1}\right)=Gini\left(y_{i}\right)-Gini({\hat{y}}_{1i})$. Each subsequent round retains the mean value of the previously used variable, then generates the mean value of a new variable, and repeats the first-round decomposition. Finally, we receive the contribution of all variables’ distributions, based on which we are able to calculate the relative contribution of each variable’s inequality to income inequality $\frac{Gini(x_{j})}{Gini(y_{i})}$. By adding up the relative contribution of all variables, the model’s explanation of income inequality can be assessed. With the semilog income generation function, we are able to compute the contribution of the residual term by subtracting the sum of contributions of all explanatory variables’ inequality from total income inequality. It should be noted that the constant term is transformed into a scalar once the estimated simlog function is solved for the original income, which can be ignored in the progress of decomposition (Shorrocks (2013) [33] provides a detailed derivation of this method on pages 4–8, and the decomposition was carried out using software provided by the UNU World Institute for Development Economics Research (UNU/WIDER)).

#### 3.1.3. Method for Mechanism Analysis

We further explore the pathways through which the happiness gap may affect income inequality by examining the effect of happiness on income in different income groups. The existing studies document that an increase in happiness would improve the individuals’ physical health [22,23], relieve stress [17,34] and increase self-improvement in spare time [30], therefore increasing income. As such, we choose three mechanism variables from CFPS, including self-rated health, mental health expressed by frequency of feeling stressed during the year, and number of hours per week spent in spare learning. The model is specified as follows:

(3) $$y_{it}=c+\beta^{\prime }{happiness}_{it}+\gamma_{i}^{\prime }X_{it}+\rho_{p}+\omega_{t}+\varepsilon_{i}$$

$y_{it}$ refers to the mechanism variables while the other variables are the same as in Equation (1). $\rho_{p}$ and $\omega_{t}$ are province-fixed effects and time-fixed effects, respectively. The definitions of other variables are the same as Equation (1). In the mechanism analysis, we divide the respondents into five groups according to their income level and conduct regressions and compare the coefficients for different income groups. We expect that different groups of people will be affected differently, which may result in income disparity between people.

### 3.2. Data

The data for our study is sourced from the China Family Panel Studies (CFPS) project. This project was conducted by the China Social Science Survey Center of Peking University, using a multistage, implicitly stratified and population-proportional sampling method. CFPS datasets contain detailed information about residents’ happiness, income, and other individual and household characteristics. We use data from five waves: 2010, 2012, 2014, 2016, and 2018. We do not utilize the CFPS 2020 and 2022 survey data due to the outbreak of the COVID-19 pandemic at the end of 2019, as it posed significant public health challenges across China, leading to large-scale emergency measures such as frequent lockdowns. These unprecedented circumstances could potentially confound our analysis, as COVID-19 has had impacts on both individual health and income inequality. Therefore, our analysis focuses on survey waves prior to the COVID-19 pandemic to avoid these confounding factors. Moreover, considering that children do not have income and that the retired population lacks wage income, we restrict our analysis to residents between 18 and 60 years old [35]. After all, we have 20,616 observations for the year 2010, 15,072 observations for 2012, 17,366 observations for 2014, 13,548 observations for 2016, and 13,667 observations for 2018 (in this study, we controlled the cognitive ability (mathematical ability and word ability) variables, which have a lot missing observations for 2014 to 2018. As a result, we have less observations from the two years).

The dependent variable in this study is the logarithm of total individual income, including wage income, operating income, transfer income, capital income and other income. Since 2014, CFPS stopped providing information on individual total income but reported household income. We refer to Berloffa and Villa (2010) and Wan (2015) [36,37] to calculate equivalent individual income for each household member by setting the weight of the household head as 1, other adult members as 0.7 and teenage and elderly members as 0.5.

The main explanatory variable of this study is “happiness”. We take Shin and Johnson’s (1978) [38] definition of happiness as “a product of the positive assessments of life situation”, we follow Specht et al. (2013) and Neumann-Böhme et al. (2021) [26,39] to measure happiness by the indicator of life satisfaction. In CFPS, respondents are asked “How are you satisfied with your life”. The answers are recorded from 1 to 5, rating respondents as “not satisfied at all”, “not satisfied”, “so-so”, “satisfied”, and “very satisfied”.

Additionally, we control for a number of individual-level characteristics in the baseline analysis, including age and age squared, education (expressed by years of education), work industry (manufacturing, low-end service industry and high-end service industry), gender (male = 1 and female = 0), hukou (urban = 1 and rural = 0) (the hukou system in China is a household registration system. For a long period, compared to rural hukou holders, urban hukou holders enjoyed more employment opportunities, better education, and healthcare services. As the economy developed, the hukou system gradually became a barrier to population movement. To address this issue, the Chinese government began to gradually advance hukou system reform at the end of the 20th century, relaxing restrictions on urban settlement and facilitating the transfer of rural population to cities), education years of the parents, migration (1 if real residence and hukou residence are different and 0 otherwise) and cognitive ability (mathematical ability and word ability). The summary statistics for the key variables are shown in Table 1.

## 4. Empirical Results

### 4.1. Descriptive Analysis

**Figure 1:**
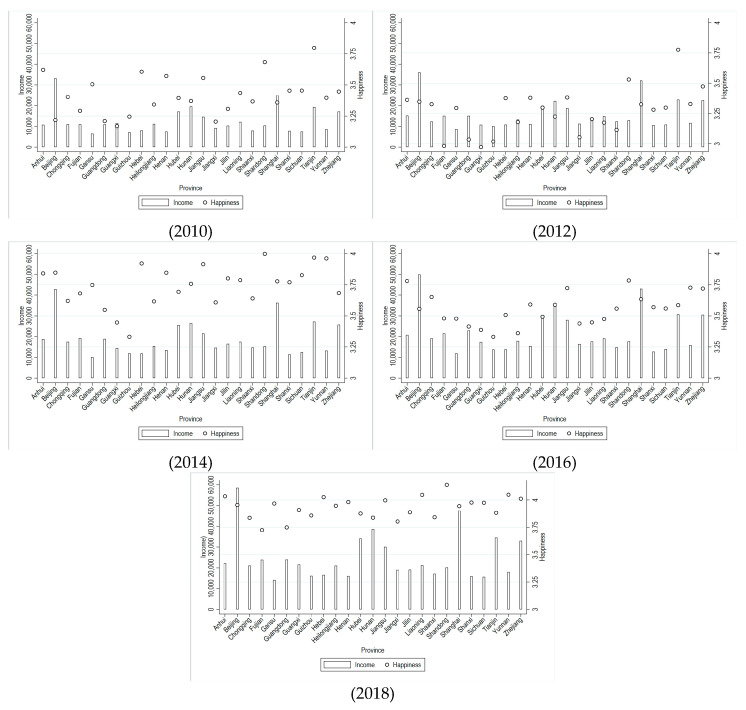
Individual income and happiness across provinces, 2010–2018. Source: CFPS.

Figure 1 depicts the average of individual income and happiness of each province for 2010, 2012, 2014, 2016, and 2018, respectively. It shows that individual income and happiness have increased over the observed period. However, provinces with the highest happiness are not necessarily the provinces with the highest income. For instance, although Beijing and Shanghai ranked the top in terms of individual income, their happiness levels stayed below the average. One reason might be that big cities like Beijing and Shanghai have strict household registration policies compared with other provinces, which prevent residents without local household registration from enjoying high-quality public services. Another reason is that big cities also have high housing prices, high consumption prices and high work pressure [40,41]. Meanwhile, there are large differences across regions. In 2010 there were significant differences in average happiness across provinces, while in 2018 the gap was narrowed.

**Figure 2:**
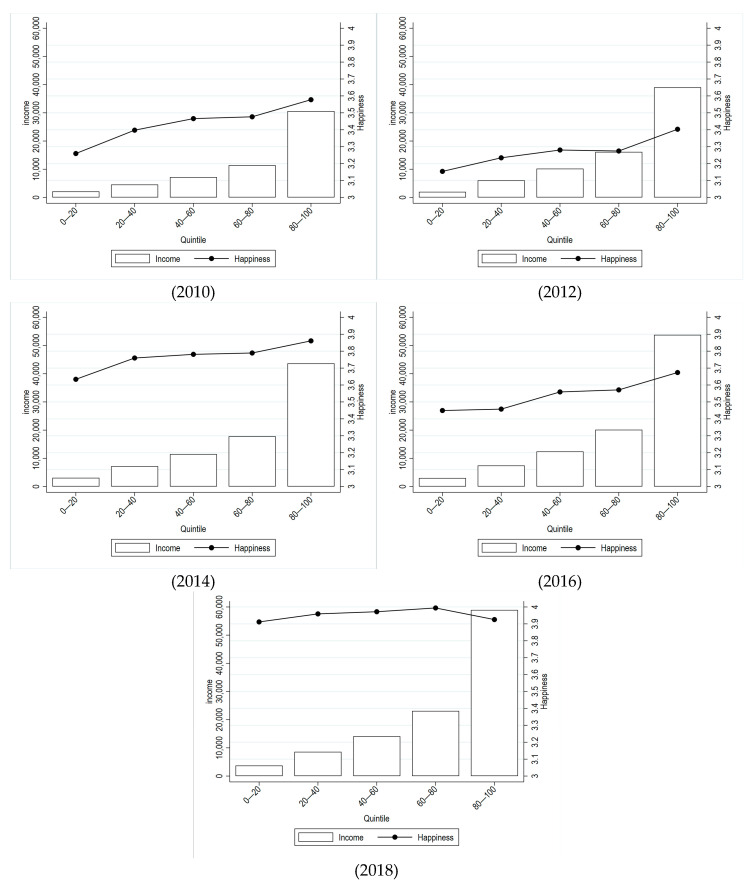
Average income and happiness among different income groups, 2010–2018. Source: CFPS.

Next, we depict individual income and happiness on average by income groups in Figure 2. It shows that there is high income disparity among different groups of people in China. The income of the top 20% respondents was 14–20 times that of the bottom 20%, about 7 times that of 20–40%, 4 times that of the 40–60%, and about 2.5 times that of the 60–80%. Additionally, we find that in general, the higher the income, the higher the happiness, implying a positive correlation between the two. We further calculate the Gini coefficient of happiness, which is 0.162, 0.167, 0.144, 0.164 and 0.129 for years 2010, 2012, 2014, 2016, and 2018, respectively, while the Gini coefficient of income for the five years is calculated to be 0.485, 0.480, 0.466, 0.471 and 0.460, respectively. These figures show that the reduction in the happiness gap may reduce income inequality.

### 4.2. Regression Results for the Income Determination Function

#### 4.2.1. Baseline Regression Results

Table 2 presents the regression results for the impact of happiness on individual income for years of 2010, 2012, 2014, 2016, and 2018 based on Equation (1). Clearly, happiness is significantly and positively connected to income. Every 1 percent increase in happiness would lead to a 0.064% to 0.124% increase in the logarithmic income.

The results for the control variables are in line with existing studies. The male has a higher possibility of obtaining higher income than the female [42]. The association between age and income presents an inverted U-shape, which is in line with [43]. Residents engaged in industries related to high-end service, manufacturing, and low-end services have similar but higher income than those in agricultural production, which is also evidenced by [14]. The income of urban residents can be 20% higher than that of rural residents. The income of residents who have migration experience or who have higher educated parents are more likely to receive higher income, which is consistent with [4]. In addition, the results provide evidence for the phenomenon of “education premium” that the income of residents with higher education is more than 25% higher than that of residents with lower education [44]. Finally, the coefficients of cognitive ability represented by mathematical ability and word ability are also significantly positive [45].

#### 4.2.2. Robustness Test

***Replacing the dependent variable.*** In the baseline regressions, we use total household income averaged by weighted household members as the dependent variable. In the robustness test, we replaced total household income with household net income, which was the total household income minus the production cost during the operating. The results are presented in Table 3, showing that using income after deducting production cost does not alter our results.

***Adding personality control variables.*** Heckman (2011) [46] proposes that personality characteristics should be considered in economic studies. Later, Cubel et al. (2016) and Attanasio et al. (2020) [45,47] confirm that personality characteristics are important predictors of career development, entrepreneurial choice and income. Hence, we include personality trait control variables in order to exclude the impact of personality factors on individual income levels.

Costa and McGrae (1992) [48] propose to divide personality into the following five aspects (big five personality traits): conscientiousness, extraversion, agreeableness, openness to experience, and neuroticism each with several subdimensions. In this study, we refer to Borghans et al. (2008) Li and Zhang (2015) [49,50] to select the 13 personality trait variables describing the above five personality aspects as control variables and re-run the regressions in Table 4. These variables are derived from the CFPS. The 13 sub-dimensions are as follows: the three sub-dimensions of conscientiousness include neatness, professionalism, and prudence; the three sub-dimensions of extraversion include enthusiasm, joyfulness, and positive emotions; the three sub-dimensions of agreeableness include trust, altruism, and submissiveness; the two sub-dimensions of openness to experience include action and value; and the two sub-dimensions of neuroticism include depression and vulnerability. Description and summary statistics for the 13 variables are presented in Table A1 and Table A2 in the Appendix A.

As is shown, the coefficients and significance for the variable of happiness do not change after adding the personality trait variables. Meanwhile, personality is also strongly associated with income. Conscientious, extroverted and open-minded individuals are likely to have higher income levels than their neurotic counterparts.

***Using CGSS dataset.*** In addition to CFPS, the China General Social Survey (CGSS) also contains information on individuals’ happiness (this study employs the China Family Panel Studies (CFPS) rather than the China General Social Survey (CGSS). The benchmark regression is that the latter has a relatively smaller sample size. The average number of valid samples per year in CGSS from 2012 to 2018 was 6280, which is only 42% of that in CFPS during the same period. Moreover, CGSS lacks information on the cities where individuals reside after 2015. Given that the China General Social Survey (CGSS) includes the question “In general, do you think your life is happy?” and has a sufficient number of valid samples each year, this study utilizes this variable to conduct a robustness check). The question is “In general, do you think your life is happy?”. The answers are scaled from 1 to 5, the higher the score, the higher the happiness. In Table 5, we regress happiness on income using CGSS, controlling for the respondents’ gender, age and age squared, education, work type, hukou, migration and parents’ education (CGSS classifies respondents’ work into entrepreneurship, self-employment, employed, and farmers. We further divided the employed into high-level employed workers and low-level employed workers, based on whether they had constant employers. Definitions for other variables are consistent with those in baseline regressions). The results are in line with the baseline regressions, which suggest that the correlation between happiness and income is significant and positive. Descriptive statistics of the variables in Table 5 are presented in Table A3 in Appendix A. 

#### 4.2.3. Endogeneity Test

Reverse causality might occur since a higher level of income can increase individuals’ happiness, causing the problem of endogeneity [51]. To address this concern, we apply the instrumental approach and use the two-stage least squares (2SLS) estimation. Specifically, we follow Bellet et al. (2024) [52] to use unexpected sunshine hours as the instrument variable of happiness, which subtracts the average daily sunshine hours of the past six years from the average daily sunshine hours of the current year. As suggested by Guven (2012) [12], people’s emotions can be influenced by the weather. Tanaka and Matsubayashi (2024) [53] also found that people are more likely to experience low mood during the winter when daylight is shorter, confirming the correlation between happiness and daylight exposure. On the one hand, the instrument variable satisfies the relevance requirement as it is positively linked to happiness, for the reason that residents facing more sunny days are more likely to report higher happiness [13]. On the other hand, the sunshine hours are unlikely to correlate with the dependent variable or with the random error term, therefore satisfying the exclusivity requirement of the instrument variable. Thus, daylight duration possesses both relevance and exogeneity, making it a suitable instrumental variable.

The 2SLS regression results are presented in Table 6. The first-stage results show that the coefficients of the instrument variable for the five years are all significantly positive, indicating that higher unexpected sunshine hours are associated with higher happiness. The F-statistics are all greater than 10, rejecting the null hypothesis of weak instrument [54]. The second-stage results indicate that happiness is still positively connected to income.

### 4.3. Decomposition Results

Based on Equations (1) and (2) we are able to calculate the Gini coefficients using the sample data. It can be seen that our calculated figures are basically consistent with the official figures, see Table 7. In addition, Table 7 shows that the overall income inequality in China declines slightly over time.

Next, we report in Table 8 the contribution of each factor’s distribution in the baseline regression to income inequality for the years of 2010, 2012, 2014, 2016, and 2018. The contribution of the happiness gap varies between 2.71% and 4.95%. On average, the happiness gap contributes 3.73 percent to total income inequality, ranking sixth. Between 2010 and 2018, the contribution of the happiness gap to income inequality decreased over time, from ranking fourth in 2010 to ranking seventh in 2018. Notably, the contribution of the happiness gap in 2018 is significantly lower than in other years. This implies that as the overall happiness increased and the happiness gap among people decreased, its contribution to income inequality would gradually decrease, thus, mitigating the rise in income disparities.

The decomposition results for other factors’ distribution are consistent with the work by Chen et al. (2011) [14]. The largest contribution comes from unobserved provincial features. There are great differences among provinces and regions in China in terms of climate, environment, culture, governance capacity, and economic development. Compared with the central and western regions, the eastern region on the one hand has rich natural and cultural resources, and on the other hand enjoys the dividends of economic reform and opening-up, thus, achieving a higher level of economic development. The contribution of work industry, years of education, and age to income inequality ranks between two and four, illustrating the importance of career choice, educational level, and work experience to income inequality. Hukou ranks fifth regarding its contribution to income inequality, indicating that the inequality in access to local public services between urban and rural areas would add to income inequality. Cognitive ability and parents’ education both contribute more than one percent to income inequality while migration accounts for less than one percent contribution.

We have also conducted the decomposition based on the 2SLS regressions, see Table 9. The results are generally robust compared with those in Table 8. The contribution of the happiness gap to income inequality and its fluctuation levels are slightly higher than the results based on the OLS regression, whilst the ranking of the happiness gap contribution basically does not change.

In Table 8 and Table 9 we use the Gini coefficient as an indicator of income inequality. We have also conducted the decomposition using inequality indicators of the Theil coefficient and the Atkinson coefficient. The decomposition results are presented in Table A4 and Table A5 in Appendix A, which are generally robust.

### 4.4. Mechanism Analysis

#### 4.4.1. The Impact of Happiness on Physical Health

Existing research has confirmed that positive emotions promote people to eat healthily, exercise more, and reduce unhealthy lifestyle habits, thereby reducing the incidence of disease and improving physical health [16,22]. Graham et al. (2004) [55] have also proved that the improvement of happiness improves health and income at the same time. Pietromonaco and Collins (2017) [56] point out that health affects residents’ interpersonal relationships, which in turn influence job opportunities. As such, we first examine the impact of happiness on physical health and test if the impact varies among different income groups. In CFPS, respondents are asked about their health status. The answers for self-rated health are recorded as 1–5, from very unhealthy to very healthy.

Table 10 shows that in general, a 1% increase in happiness can lead to a 0.106% increase in self-rated health. Yet, the impact differs across different income groups. Specifically, the marginal effect of happiness on health increases for the first four quintiles from 0.0932% to 0.1204% but decreases to 0.0992% for the fifth quintile (the group with highest income). That is, the impact of happiness on physical health presents an inverted U-shape with the increase in income. The income improvement effect on the 40–80% income groups is more obvious compared with the highest and lowest income groups.

#### 4.4.2. The Impact of Happiness on Mental Health

There has been a lot of research on how mental health may affect income and income inequality. Based on a survey study conducted in the United States, Bartel and Taubman (1986) [57] found that people with mental illness earned 8.6 percent less than the normal population. Additionally, stress exacerbates income equality and imposes long-term intergenerational effects on human capital accumulation [27]. On the other hand, mental disorders can lead to a significant decrease in annual income [58].

The CFPS surveys for 2010, 2014, and 2016 asked about the frequency of feeling stressed over the course of the year on a scale of 1 to 5, with larger numbers indicating a higher frequency of feeling stressed. The results of Table 11 confirm that the improvement of happiness is conducive to reducing the frequency of feeling stressed in residents’ lives. Overall, an increase in happiness would reduce tension frequency by 0.066%. The quintile regression results suggest that the significance for the lowest income group and the group in the 60–80% quintile disappears while the coefficient is larger for the 20–60% quintiles than that of the highest income group. The results indicate that happiness can increase income level by relieving stress, which is most significant for the middle- and low-income residents and less significant for the highest income group. Therefore, it is possible that through the mechanism of improving mental health, the contribution of happiness to income inequality is reduced. In other words, mental health is not the mechanism through which the happiness gap positively contributes to income inequality.

#### 4.4.3. The Impact of Happiness on Learning During Spare Time

Residents with high happiness are more inclined to focus on the future [12]. On the one hand, they have a strong propensity to save. On the other hand, they are more inclined to invest in their own human capital. In the process of learning, some good characteristics and habits are formed, which are conducive to the improvement of personal income, including patience [28], responsibility and ethics [59], and more prudent investment decisions [30].

In this study, we take weekly learning hours during spare time as an indicator to explore the impact of happiness on residents’ willingness to learn. In our view, learning during spare time reflects the extent to which residents can improve their cognitive ability and decision-making ability, which are essential in determining their income. The results in Table 12 indicate that happiness has no significant impact on the overall spare learning hours. But we find a significant and positive effect for the fifth quintile. As such, the differences in the impact of happiness on learning between different income groups can explain part of the income inequality among these groups.

## 5. Discussion

Figure 1 shows that, on average, happiness is positively correlated with residents’ income. However, despite South China being usually relatively more developed than North China, in 2012, 2014, and 2016, happiness’ level was higher in North China than in South China (in accordance with the Qinling-Huaihe Line for division, in our sample, South China includes provinces such as Shanghai, Jiangsu, Zhejiang, Anhui, Fujian, Jiangxi, Hubei, Hunan, Guangdong, Guangxi, Chongqing, Sichuan, Guizhou, Yunnan, while North China contains provinces such as Beijing, Tianjin, Hebei, Shanxi, Liaoning, Jilin, Heilongjiang, Shandong, Henan, Shaanxi, Gansu). This is because this relationship may also be influenced by social and cultural factors. Some research has pointed out that, the happiness, sense of achievement, and satisfaction of Asian people are not only influenced by economic factors but also directly or indirectly affected by other social factors [60]. From a socio-cultural standpoint, there exist notable disparities between Northern and Southern China. Individuals from the North are often characterized by their forthright and open-minded personalities, whereas those from the South are typically distinguished by their meticulous and detail-oriented nature. The Northern region has been profoundly shaped by Confucian cultural influences, which emphasize collectivism and strong familial bonds. In contrast, the Southern region, marked by its advanced economic development and cultural pluralism, places a greater emphasis on individualism and self-fulfillment. In addition, in families with a high value on continuing the family line, the correlation between income and happiness is stronger, whereas in families that emphasize self-actualization, the correlation is weaker [61].

Moreover, from a historical perspective, certain provinces have possessed developmental advantages, which have also been associated with relatively higher levels of happiness. For example, although the northern regions were more developed in terms of early civilization, their economic development gradually lagged behind compared to southern regions due to prolonged warfare and the southward shift in the political center. In contrast, the southern regions, with their superior natural conditions and relatively stable social environment, gradually became the economic center, resulting in relatively higher income levels and greater happiness among residents. Currently, residents in China’s eastern regions, particularly in the Yangtze River Delta and the Pearl River Delta, generally enjoy higher levels of income and happiness.

Finally, Figure 1 illustrates the positive correlation between happiness and residents’ income, a relationship that is further confirmed by the baseline results (see Table 2). This finding is also consistent with the work of [7,8], who argue that happiness can promote economic growth, as well as [24], who found that happiness can enhance labor productivity.

## 6. Conclusions

Reducing income inequality is key to realizing common prosperity and positive emotions are key to productivity or income. However, so far little is known whether income inequality will decrease if low-income individuals become happier. Based on data from CFPS, this study analyzes the impact of the happiness gap and income inequality, applying the decomposition method by Chen et al. (2011). We further explore the mechanisms through which the happiness gap may affect income inequality from perspectives of physical health, mental health, and spare time devoted to learning.

To start with, the descriptive analysis suggests that during 2010 and 2018, the happiness and income levels have both increased in China. However, in terms of the differences between different income groups, we find that the income of the top 20% is much higher than other groups. Moreover, people with a higher level of happiness usually have higher income as well.

Secondly, we examine the causal relationship between happiness and income by applying the ordinary least squares and the two-stage least squares models. The results confirm that happiness is positively and significantly associated with individual income. For every 1% increase in happiness, the logarithmic value of the level of personal income will increase by 0.064–0.124%. The results are robust by replacing the dependent variable, adding new control variables, and using different datasets.

Thirdly, we assess the contribution of the happiness gap to income inequality. Overall, the happiness gap is found to contribute between 2.71% to 4.95% to income inequality, indicating that the happiness gap among different groups of population amplifies their income inequality. Interestingly, we found that the contribution of the happiness gap to income inequality shows a downward trend as the overall happiness of residents increases. In other words, from a static point of view, the gap in happiness is a source of income inequality, while from a dynamic point of view, reducing the proportion of people who are dissatisfied with their lives will help narrow income inequality.

Fourthly, the mechanism analysis suggests that happiness has a greater impact on physical health improvement of the upper-middle-income group and, thus, contributes more to the promotion of the income of this group. While happiness does not have a significant impact on the number of hours of spare time study in the lower-middle-income group, it positively affects the highest-income group. This suggests that the happiness gap brings about income inequality mainly through promoting physical health in the middle- and high-income groups and spare learning hours in the high-income group. Interestingly, this paper also finds that happiness contributes to the decline in the frequency of psychological stress, which is stronger for the lower- and middle-income groups, which may explain why the contribution of the happiness gap to income inequality has diminished in recent years.

In addition, the results for this study have some policy implications. It is essential to enhance residents’ sense of acquisition, satisfaction, and happiness, especially the low-income group, thereby reducing income inequality. The specific policy recommendations can be made that, first, pay attention to the mental health of people with employment difficulties. Especially, the government can establish a social service and protection system that integrates skills training, employment support, and psychological treatment. Second, social organizations, social workers, and mental health experts are encouraged to actively participate in community mental health services, and attention is paid to the screening, prevention, diagnosis and treatment of mental illnesses among low-income people. Thirdly, community-based health lectures, fitness classes, and nutritional guidance can be implemented, with a focus on improving environmental sanitation facilities. Fourthly, the establishment of community learning centers that offer vocational training platforms and other forms of extracurricular learning opportunities is recommended.

Last but not least, this study also has some limitations. First, since there is currently no multidimensional scale available to measure residents’ happiness, this study can only use a single dimension, with happiness values ranging from 1 to 5. In the future, it would be possible to consider a comprehensive assessment of residents’ happiness levels from multiple dimensions, which could lead to more precise results. Second, the variables used in this study for mechanism testing are also self-reported, especially physical health and stress frequency. These variables lack objective measurements. In the future, with more accurate survey data, it would be possible to analyze which objective physical factors are affected by happiness, thereby influencing residents’ income and income inequality.

Future research may consider the following directions: first, exploring alternative decomposition methods, such as applying the Oaxaca-Blinder-RIF (OB-RIF) approach, to further decompose the structural and endowment effects of happiness. Second, using micro-level data from other countries to validate the findings and examine whether the conclusions hold globally. Third, conducting further analyses by considering the respondents’ family backgrounds and the socio-cultural characteristics of their regions to provide a more nuanced understanding of the issue.

## Figures and Tables

**Table 1:** Summary statistics for key variables.

	Mean					Std. Dev.				
	2010	2012	2014	2016	2018	2010	2012	2014	2016	2018
ln(Income)	8.73	9.50	9.79	9.90	10.11	1.08	1.29	1.03	1.05	1.19
Happiness	3.435	3.269	3.765	3.542	3.952	1.04	1.05	1.00	1.07	0.95
Age	39.7	43.1	39.7	41.6	40.9	11.8	10.5	12.0	11.2	11.7
Education	7.681	7.265	8.496	8.617	8.537	4.59	4.84	4.42	4.44	4.71
Manufacturing Employment	0.121	0.214	0.201	0.197	0.199	0.113	0.184	0.186	0.173	0.179
Low-end service Industry employment	0.303	0.295	0.363	0.378	0.376	0.298	0.284	0.305	0.286	0.291
High-end service Industry employment	0.269	0.256	0.265	0.266	0.271	0.254	0.239	0.231	0.233	0.234
Gender	0.482	0.474	0.518	0.513	0.516	0.479	0.463	0.502	0.498	0.505
Hukou	0.486	0.493	0.475	0.468	0.465	0.45	0.46	0.45	0.44	0.44
Father’s education	4.71	4.97	5.05	5.11	5.28	4.60	4.48	4.54	4.52	4.53
Mother’s education	2.77	2.60	3.05	3.08	3.32	2.03	2.33	2.11	2.59	3.21
Migration	0.263	0.320	0.228	0.228	0.194	0.073	0.114	0.105	0.116	0.129
Mathematical ability	11.2	10.8	11.6	11.6	11.4	6.55	6.30	6.28	6.18	6.11
Word ability	18.9	18.4	19.8	20.1	21.8	10.2	10.0	10.0	9.86	9.26

Source: CFPS.

**Table 2:** Results for baseline regressions.

Ln(Income)	(1)	(2)	(3)	(4)	(5)
	2010	2012	2014	2016	2018
Happiness	0.1241 ***	0.0726 ***	0.0790 ***	0.0914 ***	0.0637 ***
	(0.011)	(0.011)	(0.010)	(0.011)	(0.011)
Age	−0.0035	0.0264 ***	0.0241 ***	0.0437 ***	0.0359 ***
	(0.005)	(0.007)	(0.006)	(0.000)	(0.006)
Age squared	0.0001	−0.0005 ***	−0.0002 ***	−0.0004 ***	−0.0003 ***
	(0.000)	(0.000)	(0.000)	(0.000)	(0.000)
Education	0.0281 ***	0.0310 ***	0.0244 ***	0.0331 ***	0.0334 ***
	(0.004)	(0.005)	(0.005)	(0.004)	(0.004)
Manufacturing employment	0.2843 ***	0.6196 ***	0.2487 ***	0.2622 ***	0.2058 ***
	(0.029)	(0.033)	(0.028)	(0.033)	(0.029)
Low-end service industry employment	0.2908 ***	0.5251 ***	0.2542 ***	0.2843 ***	0.2369 ***
	(0.030)	(0.035)	(0.030)	(0.036)	(0.033)
High-end service industry employment	0.3284 ***	0.5597 ***	0.3206 ***	0.3340 ***	0.2658 ***
	(0.032)	(0.041)	(0.034)	(0.032)	(0.034)
Gender	0.0719 ***	0.1131	0.0553 ***	0.0833 ***	0.0403 **
	(0.012)	(0.020)	(0.014)	(0.015)	(0.016)
Hukou	0.1067 **	0.1176 **	0.1521 ***	0.1844 ***	0.2653 ***
	(0.043)	(0.049)	(0.034)	(0.037)	(0.033)
Father’s education	0.0048 ***	0.0125 ***	0.0095 ***	0.0079 ***	0.0130 ***
	(0.002)	(0.003)	(0.002)	(0.002)	(0.002)
Mother’s education	0.0054 ***	0.0098 **	0.0049 *	0.0124 ***	0.0099 ***
	(0.002)	(0.004)	(0.003)	(0.003)	(0.003)
Migration	0.1125 ***	0.1383 **	0.1345 **	0.0120	−0.0553
	(0.036)	(0.051)	(0.059)	(0.043)	(0.065)
Mathematical ability	0.0062 **	0.0089 ***	0.0054	0.0081 ***	0.0077 **
	(0.003)	(0.003)	(0.003)	(0.003)	(0.003)
Word ability	0.0058 ***	−0.0008	0.0047 ***	0.0031 *	0.0072 ***
	(0.002)	(0.002)	(0.002)	(0.002)	(0.002)
Constant	9.1877 ***	9.7537 ***	8.9612 ***	8.4314 ***	8.3522 ***
	(0.111)	(0.231)	(0.168)	(0.194)	(0.169)
Province FE	YES	YES	YES	YES	YES
Obs.	20,616	15,072	17,366	13,548	13,667
R^2^	0.237	0.191	0.249	0.269	0.298

Note: Robust standard errors are reported in parentheses, clustered by county; * *p* < 0.1, ** *p* < 0.05, *** *p* < 0.01.

**Table 3:** Regression results using per capita household net income.

Ln(Income)	(1)	(2)	(3)	(4)	(5)
	2010	2012	2014	2016	2018
Happiness	0.1225 ***	0.0757 ***	0.0710 ***	0.0781 ***	0.0496 ***
	(0.011)	(0.011)	(0.011)	(0.010)	(0.009)
Control variables	YES	YES	YES	YES	YES
Province FE	YES	YES	YES	YES	YES
Obs.	20,528	14,846	17,048	13,548	13,667
R^2^	0.264	0.183	0.205	0.244	0.328

Note: Robust standard errors are reported in parentheses, clustered by county; *** *p* < 0.01.

**Table 4:** Regression results by adding personality trait variables.

Ln(Income)	(1)	(2)	(3)	(4)	(5)
	2010	2012	2014	2016	2018
Happiness	0.1081 ***	0.0624 ***	0.0650 ***	0.0879 ***	0.0667 ***
	(0.010)	(0.011)	(0.010)	(0.011)	(0.011)
Neatness	0.0597 ***	0.0500 **	0.0441 ***	0.0591 ***	
	(0.018)	(0.024)	(0.013)	(0.022)	
Professionalism	0.0069				0.0372 ***
	(0.010)				(0.012)
Prudence	−0.0024	−0.0011	0.0022	−0.0301 ***	
	(0.015)	(0.009)	(0.008)	(0.010)	
Enthusiasm	−0.0186	−0.0287	0.0040	0.0357	
	(0.025)	(0.0251)	(0.016)	(0.024)	
Joyfulness	0.0011				0.0002
	(0.011)				(0.013)
Positive emotions	0.0211 *				0.0295 **
	(0.0116)				(0.012)
Trust	0.0001	0.0071	0.0112 **	0.0105 **	−0.0048
	(0.001)	(0.006)	(0.005)	(0.005)	(0.013)
Altruism	−0.1027				0.0082
	(0.010)				(0.005)
Submissiveness	0.0095	0.0399 *	−0.0275 **	0.0052	
	(0.015)	(0.022)	(0.013)	(0.016)	
Action	−0.0111	−0.0067	0.0202 *	−0.0275	
	(0.013)	(0.021)	(0.0122)	(0.015)	
Value	−0.0061		0.0196 ***		0.0062
	(0.010)		(0.012)		(0.012)
Depression	−0.0031		−0.0346 **		−0.0190 **
	(0.009)		(0.017)		(0.0085)
Vulnerability	−0.0454	−0.0276 **	−0.0298 **		
	(0.014)	(0.011)	(0.013)		
Control variables	YES	YES	YES	YES	YES
Province FE	YES	YES	YES	YES	YES
Obs.	20,409	15,028	17,334	11,587	12,663
R^2^	0.242	0.196	0.255	0.287	0.289

Note: Robust standard errors are reported in parentheses, clustered by county; * *p* < 0.1, ** *p* < 0.05, *** *p* < 0.01.

**Table 5:** Regression results by using CGSS dataset.

Ln(Income)	(1)	(2)	(3)	(4)	(5)
	2012	2013	2015	2017	2018
Happiness	0.1517 ***	0.1422 ***	0.1349 ***	0.1277 ***	0.1289 ***
	(0.017)	(0.016)	(0.019)	(0.017)	(0.024)
Control variables	YES	YES	YES	YES	YES
Province	YES	YES	YES	YES	YES
Obs.	7,016	6,616	5,494	6,198	6,076
R^2^	0.232	0.236	0.203	0.187	0.153

Note: Robust standard errors are reported in parentheses, clustered by county; *** *p* < 0.01.

**Table 6:** Two-stage least squares regression results.

	(1)	(2)	(3)	(4)	(5)
	2010	2012	2014	2016	2018
Second Stage	Dependent variable: Ln(income)				
Happiness	0.2062 **	0.1792 *	0.1083 **	0.1584 ***	0.1446 ***
	(0.1508)	(0.175)	(0.095)	(0.083)	(0.088)
Controls	YES	YES	YES	YES	YES
Province	YES	YES	YES	YES	YES
Observations	20,423	15,032	17,357	13,522	13,657
First Stage	Dependent variable: Happiness				
Sunlight hours	0.0425 ***	0.0519 ***	0.1032 ***	0.0985 ***	0.0940 ***
	(1.288)	(0.008)	(0.010)	(0.010)	(0.011)
Controls	YES	YES	YES	YES	YES
F-Value	46.79	41.15	101.65	106.15	75.54

Note: Robust standard errors are reported in parentheses, clustered by county; * *p* < 0.1, ** *p* < 0.05, *** *p* < 0.01.

**Table 7:** Gini coefficients of China.

	2010	2012	2014	2016	2018
Official Gini coefficients	0.4810	0.4740	0.4690	0.4650	0.4680
Calculated Gini coefficients	0.4846	0.4797	0.4655	0.4711	0.4597

Source: China Yearbook of Household Survey, CFPS, and own calculations.

**Table 8:** Decomposition of Gini coefficient based on the OLS regressions.

	2010			2012			2014			2016			2018			Average
	Gini	%	Rank	Gini	%	Rank	Gini	%	Rank	Gini	%	Rank	Gini	%	Rank	Rank
Happiness	0.024	4.95	4	0.015	3.11	5	0.018	3.74	6	0.019	4.12	6	0.012	2.71	7	6
Gender	0.005	1.03	10	0.009	1.84	7	0.006	1.03	9	0.001	0.56	9	0.001	0.24	9	9
Age	0.010	2.06	7	0.017	3.90	4	0.042	9.27	2	0.039	8.26	4	0.036	8.02	4	4
Education	0.044	9.07	3	0.041	8.68	3	0.041	9.06	3	0.044	9.32	3	0.048	10.42	2	3
Industry	0.046	9.48	2	0.092	19.30	1	0.041	8.91	4	0.046	9.72	2	0.038	8.19	3	2
Cognitive ability	0.015	3.09	5	0.007	1.55	9	0.011	2.01	7	0.010	2.15	7	0.013	2.86	6	7
Hukou	0.014	2.89	6	0.013	2.74	6	0.020	4.25	5	0.021	4.41	5	0.034	7.49	5	5
Migration	0.008	1.65	8	0.006	1.33	10	0.004	0.56	10	0.000	0.04	10	-0.00	-0.00	10	10
Parents’ education	0.005	1.03	9	0.008	1.81	8	0.009	1.55	8	0.007	1.45	8	0.011	2.43	8	8
Province	0.100	20.6	1	0.063	13.30	2	0.096	21.58	1	0.090	19.14	1	0.088	19.13	1	1
Residual	0.214	44.2		0.204	42.4		0.177	38.02		0.192	40.83		0.178	38.74		
Gini coefficient	0.485	100		0.475	100		0.475	100		0.471	100		0.460	100		

Note: “Happiness” represents the happiness gap or inequality. In this table, we use “Happiness” for short. Similarly, it applies to the rest variables.

**Table 9:** Decomposition results of income inequality based on the 2SLS regressions.

	2010		2012		2014		2016		2018	
	Gini	%	Gini	%	Gini	%	Gini	%	Gini	%
Happiness	0.029	5.98	0.018	3.61	0.022	4.64	0.020	4.18	0.014	2.96
Gender	0.005	1.01	0.003	0.60	0.005	1.14	0.002	0.32	0.002	0.44
Age	0.027	5.57	0.019	3.75	0.042	9.09	0.040	8.51	0.036	7.90
Education	0.044	9.03	0.039	7.98	0.041	8.76	0.042	8.92	0.048	10.49
Industry	0.045	9.34	0.091	18.38	0.042	9.02	0.047	9.93	0.037	8.05
Cognitive ability	0.014	2.89	0.006	1.28	0.011	2.45	0.010	2.17	0.014	3.00
Hukou	0.019	3.92	0.017	3.41	0.019	4.08	0.020	4.22	0.032	6.96
Migration	0.009	1.75	0.009	1.80	0.010	2.15	0.001	0.21	0.000	0.02
Parents’ education	0.004	0.91	0.016	3.21	0.009	1.80	0.009	1.85	0.011	2.35
Province	0.081	16.70	0.079	15.83	0.091	19.55	0.093	19.74	0.088	19.06
Residual	0.208	42.91	0.200	40.16	0.174	37.31	0.188	39.95	0.178	38.79
Gini coefficient	0.485	100	0.499	100	0.466	100	0.518	100	0.460	100

Note: “Happiness” represents the happiness gap or inequality. In this table, we use “Happiness” for short. Similarly, it applies to the rest variables.

**Table 10:** Results for the impact of happiness on physical health.

Health	(1)	(2)	(3)	(4)	(5)	(6)
	Total Sample	0–20%	20–40%	40–60%	60–80%	80–100%
Happiness	0.1056 ***	0.0932 ***	0.1057 ***	0.1200 ***	0.1204 ***	0.0992 ***
	(0.005)	(0.010)	(0.014)	(0.012)	(0.014)	(0.014)
Control variables	YES	YES	YES	YES	YES	YES
Province FE	YES	YES	YES	YES	YES	YES
Year FE	YES	YES	YES	YES	YES	YES
Obs.	102,969	20,584	20,595	20,595	20,587	20,600
R^2^	0.345	0.302	0.297	0.331	0.365	0.401

Note: Robust standard errors are reported in parentheses, clustered by county; *** *p* < 0.01.

**Table 11:** Results for the impact of happiness on mental health.

Mental Health	(1)	(2)	(3)	(4)	(5)	(6)
	Total Sample	0–20%	20–40%	40–60%	60–80%	80–100%
Happiness	−0.0659 ***	−0.0370	−0.0714 ***	−0.0894 ***	−0.0438	−0.0482 **
	(0.005)	(0.026)	(0.023)	(0.0266)	(0.0284)	(0.024)
Control variables	YES	YES	YES	YES	YES	YES
Province FE	YES	YES	YES	YES	YES	YES
Year FE	YES	YES	YES	YES	YES	YES
Obs.	61,771	12,323	12,368	12,363	12,357	12,356
R^2^	0.281	0.226	0.271	0.403	0.431	0.478

Note: Robust standard errors are reported in parentheses, clustered by county; ** *p* < 0.05, *** *p* < 0.01.

**Table 12:** Results for the impact of happiness on hours of spare time devoted to learning.

Learning Hours	(1)	(2)	(3)	(4)	(5)	(6)
	Total Sample	0–20%	20–40%	40–60%	60–80%	80–100%
Happiness	0.0031	−0.0008	−0.0001	−0.0114	0.0095	0.0052 **
	(0.003)	(0.006)	(0.006)	(0.011)	(0.015)	(0.002)
Control variables	YES	YES	YES	YES	YES	YES
Province FE	YES	YES	YES	YES	YES	YES
Year FE	YES	YES	YES	YES	YES	YES
Obs.	44,938	9010	8965	8960	8988	9011
R^2^	0.069	0.039	0.052	0.113	0.108	0.169

Note: Robust standard errors are reported in parentheses, clustered by county; ** *p* < 0.05.

## Data Availability

The data that support the findings of this study are available from the corresponding author upon reasonable request.

## Appendix A

### Definitions of Variables in Table 1

Ln(income) is a logarithm of individual income, including wage income, operating income, transfer income, capital income, and other income. It is calculated based on household income using the equivalent scale Wan (2015). The equivalent scale is that we set the weight of the household head as 1, other adult members as 0.7, and teenage and elderly members as 0.5.

Happiness is the rate of “How are you satisfied with your life”. It ranges from 1 to 5, rating respondents as “not satisfied at all”, “not satisfied”, “so-so”, “satisfied”, and “very satisfied”.

Regarding other variables, age is current year minus the year of birth; education is measured by years of education; work industry is classified into agriculture, manufacturing and services, according to “Industrial Classification for National Economic Activities” established by the National Bureau of Statistics of China. We also refer to Bárány and Siegel (2018) and Tang and Jiang (2022) [62,63] to categorize the service sector into low-end and high-end services industry based on the educational level of the workforce. Therefore, we use three dummy variables to represent respondents’ employment: manufacturing employment, low-end service industry employment, and high-end service industry employment. If the respondent’s industry falls into one of these categories, the corresponding dummy variable is assigned a value of 1, otherwise, it is assigned a value of 0.

Gender is a dummy variable, where male = 1 and female = 0; hukou is a dummy variable, where urban = 1 and rural = 0; education years of the parents is expressed by years of education of interviewees’ parents; migration is a dummy variable, equals to 1 if real residence and hukou residence are different and 0 otherwise.

Cognitive ability is sourced from tests administered to respondents in the CFPS survey. In the CFPS survey, all individuals aged 10 and above undergo cognitive testing during face-to-face interviews to reflect the respondents’ educational achievement and potential ability. The theoretical basis for the CFPS math test (mathtest) and word test (wordtest) is the Guttman Scale from psychometrics. The math test scores range from 0 to 24, while the word test scores range from 0 to 34).

**Table A1:** CFPS personality trait variables and their definitions.

Dimensions	Sub-Dimensions	Definition or Characteristic	Questions in CFPS
Conscientiousness	Neatness	Handling affairs in an orderly manner	Cleanliness of clothing
	Professionalism	Initiative in achieving goals	Importance of having a sense of accomplishment
	Prudence	Prudence before taking concrete actions	Doubts about the survey
Extroversion	Enthusiasm	Attitude towards others and relationships	Level of hospitality
	Joyfulness	Willingness to be a partner to others	Importance of not being alone
	Positive emotions	Have fun in life	Importance of having fun in life
Agreeableness	Trust	Trust to others	Level of trust to strangers
	Altruism	Level of attention to others’ interests and needs	Importance of not being hated
	Submissiveness	Willingness to cooperate with others	Degree of cooperation with the survey
Openness	Action	Tendency to try different activities	Interest to the survey
	Value	Degree of support for traditional values	Importance of succession
Neuroticism	Depression	Feeling frustration	Frequency of frustration
	Vulnerability	Feeling vulnarable	Frequency of feeling hopeless about the future

Source: CFPS.

**Table A2:** Mean values of CFPS personality trait variables.

	2010	2012	2014	2016	2018
Neatness	5.1288	5.3346	5.6571	5.7524	
Professionalism	3.7484				3.8734
Prudence	2.5792	2.2898	2.3818	2.2706	
Enthusiasm	5.1930	5.3611	5.6900	5.8788	
Joyfulness	4.0334				4.1498
Positive emotions	4.0630				4.1560
Trust	3.9801	2.0975	1.9848	2.0758	2.3650
Altruism	5.5452		5.8012		3.9481
Submissiveness	4.9572	5.5791	5.4140	5.9762	
Action	3.9620	4.9948	3.2374	5.5982	
Value	4.4453		4.3756		4.0533
Depression	4.2772		4.2141		3.1094
Vulnerability	4.6574	1.4145	4.6816		

Source: CFPS. Note: Among the above 13 variables, neatness, prudence, enthusiasm, altruism, and submissiveness take values in the range of 1–7, and the rest take values in the range of 1–5.

**Table A3:** Mean values of CGSS variables.

	2012	2013	2015	2017	2018
Ln(Income)	10.06	10.19	10.52	10.74	10.81
Happiness	3.80	3.75	3.84	3.82	3.97
Gender	0.51	0.51	0.47	0.47	0.47
Age	41.64	41.30	41.50	41.60	41.90
Education	9.54	9.72	9.74	10.21	9.97
Agricultural employment	0.24	0.23	0.20	0.18	0.17
Low-level employee	0.32	0.34	0.39	0.40	0.40
High-level employee	0.34	0.33	0.31	0.33	0.33
Boss	0.01	0.01	0.02	0.02	0.03
Self-employed	0.09	0.10	0.07	0.07	0.07
Household registration status	0.43	0.39	0.36	0.23	0.25
Migration	0.11	0.11	0.11	0.09	0.10
Father’s education	5.61	5.65	5.20	6.01	5.89
Mother’s education	3.72	3.87	3.73	4.28	4.24

Souce: CGSS. Note: Variables in Table A3 include Ln(Income), which is the logarithm of individual income; happiness is the self-rated happiness of interviewees; education is expressed by years of education; agricultural employment, boss, self-employment, low-level employed employees, and high-level employed workers represent working status; gender is a dummy variable (male = 1 and female = 0); household registration status is a dummy variable (urban = 1 and rural = 0); father’s and mother’s education means education years of the parents; migration is a dummy variable (1 if real residence and hukou residence are different and 0 otherwise).

**Table A4:** Decomposition of the Theil index based on the OLS regressions.

	2010		2012		2014		2016		2018	
	Theil	%	Theil	%	Theil	%	Theil	%	Theil	%
Happiness	0.008	1.80	0.007	1.53	0.003	0.78	0.005	1.23	0.003	0.72
Gender	0.000	0.00	−0.001	−0.15	0.001	0.25	0.000	0.00	0.000	−0.03
Age	0.001	0.32	0.005	1.19	0.005	1.28	0.002	0.53	0.009	2.16
Education	0.017	3.95	0.023	5.25	0.017	4.67	0.020	4.74	0.024	5.96
Industry	0.019	4.29	0.052	12.10	0.017	4.60	0.022	5.01	0.016	3.98
Cognitive ability	0.006	1.35	0.004	0.90	0.003	0.86	0.005	1.17	0.007	1.68
Household registration	0.006	1.32	0.007	1.69	0.007	2.01	0.011	2.54	0.018	4.48
Migration	0.003	0.69	0.003	0.74	0.001	0.31	0.000	0.02	0.000	−0.03
Parents’ education	0.002	0.35	0.004	0.96	0.002	0.59	0.003	0.69	0.005	1.27
Province	0.037	8.47	0.033	7.59	0.047	12.78	0.051	11.85	0.048	11.91
Residual	0.341	77.70	0.294	68.20	0.263	71.86	0.312	72.22	0.273	67.90
Theil index	0.438	100.00	0.431	100	0.366	100	0.432	100	0.402	100

Note: This table presents the results of the decomposition of the Theil index based on the baseline regression results, following the method of [14]. Contributions of all variables to the Theil index are presented. For example, happiness accounts for 0.72–1.80% of the total income inequality (measured by Theil index) from 2010 to 2018.

**Table A5:** Decomposition of the Atkinson’s index based on the OLS regressions.

	2010		2012		2014		2016		2018	
	Atkinson	%	Atkinson	%	Atkinson	%	Atkinson	%	Atkinson	%
Happiness	0.006	2.86	0.002	1.12	0.002	1.42	0.003	1.32	0.003	1.44
Gender	0.000	0.00	0.000	0.13	0.000	0.26	0.000	0.01	0.000	0.01
Age	0.001	0.58	0.002	1.20	0.006	3.70	0.003	1.67	0.002	1.26
Education	0.009	4.32	0.011	5.39	0.008	4.89	0.010	5.13	0.010	5.59
Industry	0.009	4.65	0.025	12.4	0.008	4.82	0.010	5.34	0.010	5.82
Cognitive ability	0.003	1.42	0.002	0.91	0.001	0.88	0.002	1.25	0.002	1.36
Household registration	0.005	2.43	0.004	1.77	0.004	2.13	0.005	2.74	0.005	2.98
Migration	0.002	0.76	0.002	0.79	0.001	0.34	0.000	0.02	0.000	0.02
Parents’ education	0.002	0.88	0.002	0.99	0.001	0.62	0.001	0.75	0.001	0.82
Province	0.019	9.41	0.017	8.24	0.023	13.78	0.026	13.16	0.026	14.35
Residual	0.147	72.68	0.135	67.33	0.113	67.16	0.134	68.63	0.119	66.35
Atkinson’s index	0.202	100	0.201	100	0.168	100	0.195	100	0.179	100

Note: This table presents the results of the decomposition of the Atkinson’s index based on the baseline regression results, following the method of [14]. Contributions of all variables to the Atkinson’s index are presented. For example, happiness accounts for 1.12–2.86% of the total income inequality (measured by the Atkinson’s index) from 2010 to 2018.

**Table A6:** Summary statistics for the mechanism variables.

	Variables	Mean	Std.Dev.	Min	Max
Total sample	Health	2.6496	1.2536	1	5
	Frequency of tension	4.0700	1.1157	1	5
	Spare time learning hours	0.0759	0.3456	0	10
0–20%	Health	2.8005	1.3520	1	5
	Frequency of tension	4.0080	1.1287	1	5
	Spare time learning hours	0.0397	0.2574	0	6
20–40%	Health	2.6918	1.2852	1	5
	Frequency of tension	4.0662	1.1171	1	5
	Spare time learning hours	0.0497	0.2708	0	6
40–60%	Health	2.6283	1.2570	1	5
	Frequency of tension	4.0838	1.1018	1	5
	Spare time learning hours	0.0609	0.3139	0	8
60–80%	Health	2.5819	1.2074	1	5
	Frequency of tension	4.0897	1.1118	1	5
	Spare time learning hours	0.0890	0.3743	0	10
80–100%	Health	2.5453	1.1403	1	5
	Frequency of tension	4.1018	1.1166	1	5
	Spare time learning hours	0.1396	0.4605	0	7.14

Source: CFPS.
